# Risk Taking for Potential Reward Decreases across the Lifespan

**DOI:** 10.1016/j.cub.2016.05.017

**Published:** 2016-06-20

**Authors:** Robb B. Rutledge, Peter Smittenaar, Peter Zeidman, Harriet R. Brown, Rick A. Adams, Ulman Lindenberger, Peter Dayan, Raymond J. Dolan

**Affiliations:** 1 Max Planck University College London Centre for Computational Psychiatry and Ageing Research, London WC1B 5EH, UK; 2 Wellcome Trust Centre for Neuroimaging, University College London, London WC1N 3BG, UK; 3 Center for Lifespan Psychology, Max Planck Institute for Human Development, 14195 Berlin, Germany; 4 European University Institute, San Domenico di Fiesole, 50014 Fiesole, Italy; 5 Gatsby Computational Neuroscience Unit, University College London, London W1T 4JG, UK

## Abstract

The extent to which aging affects decision-making is controversial. Given the critical financial decisions that older adults face (e.g., managing retirement funds), changes in risk preferences are of particular importance [1]. Although some studies have found that older individuals are more risk averse than younger ones [2, 3, 4], there are also conflicting results, and a recent meta-analysis found no evidence for a consistent change in risk taking across the lifespan [5]. There has as yet been little examination of one potential substrate for age-related changes in decision-making, namely age-related decline in dopamine, a neuromodulator associated with risk-taking behavior. Here, we characterized choice preferences in a smartphone-based experiment (n = 25,189) in which participants chose between safe and risky options. The number of risky options chosen in trials with potential gains but not potential losses decreased gradually over the lifespan, a finding with potentially important economic consequences for an aging population. Using a novel approach-avoidance computational model, we found that a Pavlovian attraction to potential reward declined with age. This Pavlovian bias has been linked to dopamine, suggesting that age-related decline in this neuromodulator could lead to the observed decrease in risk taking.

## Highlights

• Aging reduced risk taking for potential gains but not potential losses
• Computational models revealed that a Pavlovian influence of reward decreased with age
• Age-related dopamine decline can explain the decrease in Pavlovian biases

## Teaser

Rutledge et al. used a large-scale smartphone-based experiment to investigate how aging affects decision-making under uncertainty. Risk taking for potential rewards but not losses gradually decreased over the lifespan, consistent with an explanation in terms of age-related decline in the dopamine system.

## Results

Risk and reward are tightly coupled, justifying the attention to understanding risk across various fields. Pavlovian influences are of particular significance for understanding anomalies of choice. We recently identified one such anomaly with the finding of a pervasive tendency to approach potential reward and avoid potential punishments irrespective of option value [6]. This effect is distinct from parametric decision models based on prospect theory [7] that operationalize concepts like risk and loss aversion [8, 9, 10, 11]. One important modulator of appetitive Pavlovian influences is dopamine. We found that boosting dopamine levels with levodopa (L-DOPA) dose-dependently amplified the Pavlovian influence of potential reward, increasing risk taking in situations with potential gains but not losses [6]. This finding may account for why electrical [12] and optogenetic [13, 14] stimulation of dopamine neurons increases reward seeking and why dopamine drugs increase risk taking [15] and pathological gambling in Parkinson’s patients [16].

A profound change in the aging brain is a gradual decline in the integrity of the dopamine system, corresponding to a likely functional loss of dopamine. Dopamine receptor and transporter densities decrease at rates estimated for many brain areas at up to 10% per decade throughout the adult lifespan [17, 18, 19]. These neuromodulatory changes are linked to cognitive changes [20, 21, 22] and changes in neural responses to reward [23, 24, 25]. Given the link between dopamine and risk taking, as well as the critical significance of risk in financial decisions such as saving for retirement, it becomes pressing to understand the relationship between age and risk taking. Previous investigations have been somewhat equivocal [5]. However, whether aging affects Pavlovian influences on choice has not been previously investigated.

We made several predictions for how decision-making under uncertainty would be affected by aging. First, the number of risky options chosen in trials with potential gains but not losses would decrease with age, consistent with a finding that boosting dopamine levels increases risk taking for potential gains but not losses. Second, age-related decline in risk taking would be monotonic, reflecting the gradual age-related decline in the dopamine system. Finally, Pavlovian approach parameters capturing a tendency to choose risky options with potential reward would decrease with age and be more strongly associated with aging than risk-aversion parameters in standard models. A standard clinical dose of 150 mg of L-DOPA increased choice to risky options with potential reward by 5% on average [6]. Given this modest effect, addressing the prediction that aging decreases risk taking for potential reward requires a sample size far greater than is typical for laboratory studies. Even a small effect size [26] (e.g., r = 0.1) would have important economic consequences given the large and growing elderly population [27].

**Figure 1:**
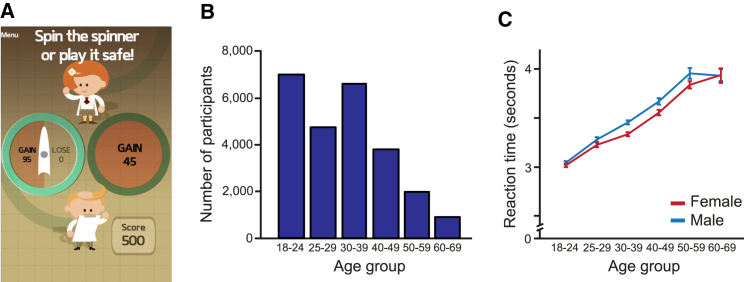
Task Design (A) Gain trials have only potential gains and not potential losses. In an example gain trial, a participant chooses between a risky option (here, a potential reward of 95 points) and a safe option (here, gaining 45 points). Mixed trials have both potential gains and losses. Loss trials have only potential losses and not potential gains. (B) Participants self-identified into age bands, with 2,945 participants aged 50 and older. (C) Decision reaction times increased with age for both females and males. Error bars represent the SEM.

To test our predictions, we utilized a smartphone-based platform (The Great Brain Experiment, http://www.thegreatbrainexperiment.com), freely available for Apple iOS and Google Android systems [28, 29, 30]. We collected a dataset with a sample size (n = 25,189) many times larger than all previous laboratory studies on aging and decision-making under uncertainty combined (meta-analysis in [5]). Participants completed a 30-trial game in which they tried to earn points. On each trial, participants faced a choice between safe and risky options with no time limit to make their decisions (Figure 1A; see the Experimental Procedures). Risky options were represented by spinners with equal probabilities for two potential outcomes, and chosen gambles were resolved immediately. There were three types of trials: (1) gain trials, a certain gain or a gamble with a larger potential gain or zero; (2) mixed trials, zero or a gamble with a potential gain or loss; and (3) loss trials, a certain loss or a gamble with a larger potential loss or zero. Importantly, losses were not possible in gain trials and gains were not possible in loss trials, allowing us to dissociate effects of age on risk taking in gain and loss domains. The average player earned 580 points (starting from an endowment of 500 points), greatly exceeding the 514 points a random player earns on average. A player that always chooses the option with the higher expected value earns 678 points on average and chooses the risky option in 54% of trials (gain, 57%; mixed, 65%; loss, 43%). Data were analyzed for six different age groups spanning the range of 18–69 with the oldest group (ages 60–69) containing 931 participants (Figure 1B). As expected, median decision reaction times (Figure 1C) increased with age in both males and females (r = 0.22, p < 0.001; all p values were computed by permutation test; see the Experimental Procedures). Participants on average chose the risky option in 64% of trials (gain, 69%; mixed, 67%; loss, 56%).

Age-related changes in the brain have been proposed to lead to increased noise in either value representations or choice mechanisms [31], either of which could reduce the consistency with which participants make the same choice when faced with similar options on multiple occasions. Participants were overall consistent in their choices, making the same choice 70% of the time when offered similar options in two different trials (see the Supplemental Experimental Procedures). There was no age-related change in choice consistency (r = 0.001, p > 0.1), arguing against the theory that increased noise might lead to more frequent errors, at least for simple economic decisions.

**Figure 2:**
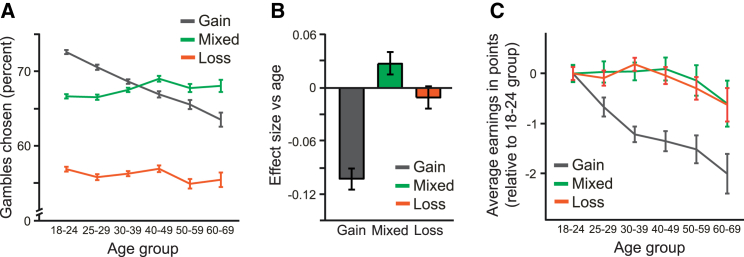
Risk Taking for Potential Reward Decreased across the Lifespan (A) The percentage of trials in which risky options were chosen was relatively stable for mixed and loss trials but declined steadily over the lifespan in gain trials. Young participants chose more risky options in gain than mixed trials, whereas the opposite was true for older participants. Error bars represent the SEM. (B) The age-related decline in risk taking in gain trials had an effect size equal to −0.103. Risk taking did not decrease across the lifespan in trials with potential losses (mixed or loss trials). Error bars represent bootstrapped 95% confidence intervals. (C) Compared to the youngest age group (18–24), earnings decreased significantly with age in gain trials, but not in mixed or loss trials. Error bars represent the SEM.

Next, we tested whether risk-taking behavior changed across the lifespan, starting by examining trials with potential losses (Figure 2A). There was a small but significant increase in risk taking in trials with both potential gains and losses (mixed trials: r = 0.024, p < 0.001; Figure 2B). However, this change was not monotonic, as would be expected if it resulted from gradual age-related decline in dopamine. There was no change in risk taking in trials with only potential losses (loss trials: r = −0.010, p > 0.1). There was also no significant change in earnings across the lifespan in mixed (r = −0.004, p > 0.1) and loss trials (r = −0.008, p > 0.1; Figure 2C).

By contrast, there was a substantial decline in the number of risky choices made in gain trials, which featured potential gains but not potential losses (Figure 2A). This age-related change was gradual and monotonic, with all age groups making fewer risky choices on average than the next younger age group (all five pair-wise comparisons, p < 0.001 by permutation test after Bonferroni correction). This monotonicity was present in only 0.4% of 10,000 resamples (see the Experimental Procedures). The effect size for a linear model of decline was r = −0.103 (p < 0.001; Figure 2B), significantly greater than effect sizes in mixed or loss trials (both comparisons, p < 0.001). Furthermore, earnings in gain trials significantly declined over the lifespan (r = −0.045, p < 0.001; Figure 2C), suggesting that the observed age-related changes in decision-making may have important economic consequences. We found a similar decline in risk taking in gain trials in both the United Kingdom (n = 10,300, r = −0.103) and the United States (n = 2,361, r = −0.098), the two countries for which we had sufficiently large samples. After excluding participants in the youngest age group (who may not yet have completed university education), we found a similar decline in participants with (n = 11,965, r = −0.082) and without (n = 6,194, r = −0.074) a university degree. We also found a similar decline for Apple iOS (n = 14,305, r = −0.098) and Google Android (n = 10,884, r = −0.110) smartphones.

In a study examining the effect of pharmacologically boosting dopamine [6], we previously described a model that included three components: (1) loss aversion (parameter λ) and risk aversion in gain and loss domains (α_gain_ and α_loss_) according to established parametric models based on prospect theory [8, 9, 10, 11], (2) stochasticity of decision-making according to the inverse temperature parameter (μ) in the softmax equation, and (3) Pavlovian approach-avoidance parameters applying exclusively to gain trials (β_gain_) or loss trials (β_loss_). Boosting dopamine affected only the Pavlovian influence of potential reward (β_gain_). Based on the observation that the dopamine system declines gradually over the lifespan, we tested the prediction that this factor would underpin the observed decline in gambling. The model-based approach permits testing which of the three model components (α_gain_, μ, or β_gain_) is most strongly associated with age.

This approach-avoidance decision model fitted choice data from the smartphone sample (pseudo-r^2^ = 0.46 ± 0.25, mean ± SD; see the Experimental Procedures) better than an established decision model based on prospect theory according to Bayesian model comparison [32, 33] (lower Bayesian information criterion [BIC] preferred: approach-avoidance decision model BIC = 12,737 versus prospect theory decision model BIC = 13,620).

Consistent with the observation above that choice did not get noisier, we found that the stochasticity parameter μ did not change over the lifespan (males, r = 0.010; females, r = −0.004). Loss aversion might be expected to increase with age, but we found, if anything, evidence for a small decrease (males, r = −0.015; females, r = −0.026). Average loss-aversion coefficients (males, λ = 1.91; females, λ = 1.85) and risk-aversion coefficients (males, α_gain_ = 0.86 and α_loss_ = 0.70; females, α_gain_ = 0.82 and α_loss_ = 0.63) were comparable to those reported in prior studies [4, 9].

**Figure 3:**
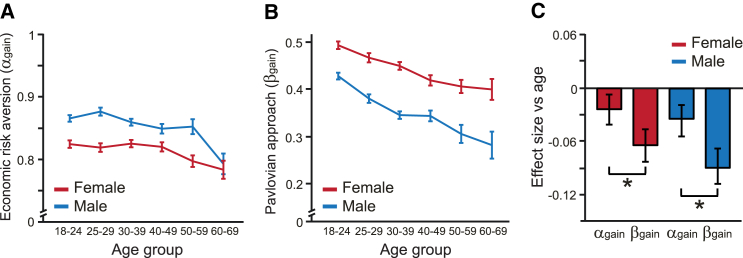
Pavlovian Approach for Potential Reward Decreased across the Lifespan (A and B) Model fits in both females and males showed modest declines in economic risk-aversion parameters (α_gain_; A) and larger declines in Pavlovian approach parameters (β_gain_; B). Error bars represent the SEM. (C) For both females and males, effect sizes for age-related decline in Pavlovian approach parameters were larger in magnitude than for economic risk-aversion parameters. Error bars represent bootstrapped 95% confidence intervals. ^∗^p < 0.001.

Increasing age was accompanied by modest decreases in economic risk-aversion parameters (α_gain_; Figure 3A) in both males (r = −0.034) and females (r = −0.024). Since risk-aversion parameters less than 1 correspond to risk aversion, these modest decreases are consistent with the overall pattern of decreased risk taking with age. As we predicted, there was a highly significant age-related decrease in Pavlovian approach parameters (β_gain_; Figure 3B) in both males (r = −0.090) and females (r = −0.064). Effect sizes for the relationship between β_gain_ and age were more negative than effect sizes for α_gain_ (both males and females, p < 0.001; Figure 3C), with an average effect-size ratio in males of 2.69 (bootstrapped 95% confidence interval, 1.39–9.29) and an average effect-size ratio in females of 2.60 (bootstrapped 95% confidence interval, 1.56–5.70). Thus, we find that advancing adult age is more strongly associated with a decreased Pavlovian influence of potential reward than a decrease in a risk-aversion parameter of standard decision models based on prospect theory.

## Discussion

Normal human aging affects many cognitive abilities [22, 34]. Given the increasing size of the global elderly population, understanding how aging affects economic decision-making is of critical importance. Using a smartphone-based methodology, we collected a sample (n = 25,189) much larger than that of all previous laboratory studies on decision-making under uncertainty combined [5]. We observed a substantial decrease in the number of risky options chosen in trials with potentials gains but not losses. We observed a preference reversal by which younger participants were more likely to choose risky options in gain than mixed trials and the opposite for older participants. Two possible explanations for risk-taking changes were increased errors or decreased risk-aversion parameters [4]. However, aging did not affect choice consistency and had only a modest effect on risk-aversion parameters. We recently reported that boosting dopamine with L-DOPA increased the Pavlovian influence of potential reward [6]. Such immediate dopaminergic effects are not explained by the established role of dopamine in learning [35, 36, 37, 38]. Given the widespread gradual decline in the integrity of the dopamine system across the lifespan [17, 18, 19], we hypothesized that normal cognitive aging would reduce the Pavlovian influence of potential reward. Using a model-based analysis, we found that adult age is more strongly associated with a decrease in the Pavlovian influence of potential reward than with a decrease in risk-aversion parameters.

An ideal player who always selects the option with the higher expected value chooses the risky option in 57% of the gain trials on average. If economic risk-aversion parameters are equal to 1, positive Pavlovian approach parameters can reduce average earnings. Economic risk-aversion parameters were on average less than 1 in all age groups, reflecting a concave utility function that leads to risk aversion and reduced earnings. In this case, positive Pavlovian approach parameters can increase earnings, explaining why age-related decreases in this parameter are associated with decreased earnings. Whether positive Pavlovian parameters increase or decrease earnings also depends on the available options. If risky options in most trials are worth less than certain alternatives, a decrease in Pavlovian parameters could actually increase earnings. The economic implications of decreased Pavlovian parameters depend on situations routinely faced by older individuals. Most financial investments feature both potential gains and losses. Simple economic decisions in this domain may be suboptimal due to loss aversion but relatively unaffected by aging. However, financial options might be presented in such a way as to appear similar to gain trials, and in this situation older individuals might choose risky options less frequently than younger individuals even if they yield greater returns on average.

Smartphone-based data are more representative of the overall population than conventional laboratory experiments [39, 40]. 85% of users in the local university subject pool are currently university students. In contrast, 73% of our participants are age 25 or older, and 42% report not having a university degree.

Given the many factors likely to contribute to economic preferences, addressing our hypothesis required a very large sample. It would have been surprising to observe effect sizes larger than those we found (r = ∼0.1) due to a single factor such as age-related dopaminergic decline. In trials with potential gains and not losses, we observed an 8% decrease in risk taking from the youngest to the oldest age group, even larger than we anticipated given the 5% increase observed in young volunteers after taking L-DOPA [6]. Sample sizes in previous studies [5] may not have been sufficiently large to identify age-related changes. Task design may also play an important role in determining the size of Pavlovian influences. Pavlovian approach parameters in participants playing monetary lotteries were smaller than those observed here with unpaid participants [6]. Conflicting results reported in a recent meta-analysis [5] may reflect the varying degree to which risk taking in different tasks depends on Pavlovian influences.

In the domains of episodic memory [41] and working memory [42], pharmacological interventions with dopaminergic drugs can restore youth-like brain activation patterns and behavior in healthy older adults. Understanding the role of dopamine in decision-making is particularly important when it comes to addressing the unintended side effects of dopaminergic drugs, such as those commonly prescribed to individuals suffering from Parkinson’s disease, schizophrenia, and attention deficit hyperactivity disorder. For simple economic decisions, we found no decrease across the lifespan in either choice consistency or risk taking for potential losses. This selective pattern of changes is hard to reconcile with general explanations unrelated to normal cognitive aging, such as cohort differences in familiarity with computerized games. However, this pattern of results is fully consistent with an explanation in terms of age-related dopaminergic decline. At the population level, knowing the specific choice situations in which aging does and does not affect decision-making may be useful for policymakers, and the existence of these age-related changes in behavior may have important economic consequences.

## Experimental Procedures

### Participants

We tested 25,189 participants (aged 18–69, 11,951 male) who completed the task between May 1, 2013 and September 1, 2014. All participants gave informed consent, and the Research Ethics Committee of University College London approved the study.

### Smartphone-Based Experiment

Participants completed 30 choice trials and rated their happiness 12 times, typically in 3–5 min. An analysis of happiness responses has been reported previously [29]. Consistent with previous research [43], happiness increased with age (Supplemental Experimental Procedures). Each play consisted of 11 gain, 8 mixed, and 11 loss trials (Supplemental Experimental Procedures).

### Data Analysis

We analyzed the first play from each participant (Supplemental Experimental Procedures). We report Pearson correlation coefficients for effect sizes of relationships between task measures and age. All p values were computed based on permutation tests using 10,000 random shuffles of age labels to determine null distributions. Bootstrapped 95% confidence intervals were computed based on 10,000 resamples with replacement in each age or age/gender group. We fitted choices in individual participants with an approach-avoidance decision model [6]. As in common parametric decision models [4, 8, 9, 10, 11], subjective values or utilities were determined as follows:

$$U_{gamble}=0.5{\left(V_{gain}\right)}^{\alpha_{gain}}-0.5\lambda {\left(-V_{loss}\right)}^{\alpha_{loss}},$$

$$U_{certain}={\left(V_{certain}\right)}^{\alpha_{gain}}\;if\;V_{certain}\geq 0,$$

and

$$U_{certain}=-\lambda {\left(-V_{certain}\right)}^{\alpha_{loss}}\;if\;V_{certain}<0,$$

where *V*_*gain*_ and *V*_*loss*_ are the potential gain and loss from a gamble, respectively, and *V*_*certain*_ is the certain option value. Choice probabilities are commonly determined by the softmax rule:

$$P_{gamble}=\frac{1}{1+e^{-\mu \left(U_{gamble}-U_{certain}\right)}},$$

where the inverse temperature parameter μ quantifies choice stochasticity. We modified this equation to permit choice probabilities that differ from 0 or 1 in the limit. For gain trials, gambling probability was determined by β_gain_:

$$P_{gamble}=\frac{\left(1-\beta_{gain}\right)}{1+e^{-\mu \left(U_{gamble}-U_{certain}\right)}}+\beta_{gain}\;if\;\beta_{gain}\geq 0$$

and

$$P_{gamble}=\frac{\left(1+\beta_{gain}\right)}{1+e^{-\mu \left(U_{gamble}-U_{certain}\right)}}\;if\;\beta_{gain}<0.$$

Model parameters were fit by the method of maximum likelihood in individual participants, and we used Bayesian model comparison techniques [32, 33] to compare model fits.

## Author Contributions

R.B.R., U.L., P.D., and R.J.D. designed the research. R.B.R., P.S., P.Z., H.R.B., and R.A.A. contributed to app development. R.B.R. analyzed the data. All authors contributed to writing the paper.

## Supplemental Information

Document S1. Supplemental Experimental Procedures

Document S2. Article plus Supplemental Information
