# Supplementary material for: Risk Taking for Potential Reward Decreases across the Lifespan
Source: Curr Biol. 2016 Jun 20;26(12):1634–9. doi: 10.1016/j.cub.2016.05.017 (PMC4920952; doi:10.1016/j.cub.2016.05.017)
Supplement: Document S1. Supplemental Experimental Procedures [file mmc1.pdf]

Current Biology, Volume *26*

Supplemental Information

Risk Taking for Potential Reward

Decreases across the Lifespan

Robb B. Rutledge, Peter Smittenaar, Peter Zeidman, Harriet R. Brown, Rick A. Adams, Ulman Lindenberger, Peter Dayan, and Raymond J. Dolan

Supplemental Experimental Procedures

Experimental Task Design

Trials were randomly drawn from lists of 60 gain, 30 mixed, and 60 loss trials. The gain trial list consisted of 4 certain amounts {30, 35, 45, 55} with gamble gain amounts determined by 15 multipliers on the certain amount to accommodate a wide range of risk sensitivity {1.64, 1.7, 1.76, 1.82, 1.88, 1.94, 2, 2.06, 2.12, 2.18, 2.26, 2.4, 2.7, 3.2, 4}. The mixed trial list consisted of 3 gamble gain amounts {40, 55, 75} and gamble loss amounts determined by 10 multipliers on the gain amount to accommodate a wide range of loss sensitivity {0.2, 0.34, 0.5, 0.64, 0.77, 0.89, 1, 1.1, 1.35, 2}. The loss trial list consisted of 4 certain amounts {-30, -35, -45, -55} and gamble loss amounts determined by the same 15 multipliers as gain trials. The maximum gain or loss possible from a single trial was 220 points, and participants started the game with an endowment of 500 points. Participants were presented with the question 'How happy are you right now?' after every 2-3 trials. Participants indicated their responses on a rating line and pressed a button labeled 'continue' to proceed to the next trial. Participants were informed of their current earnings during all choice trials. Each play started and ended with a happiness question.

An analysis of happiness responses during the task has been reported previously [S1]. Consistent with previous research [S2], we found that life satisfaction measures (collected when participants first downloaded the app) increased with age ($r = 0.083$, $p < 0.001$), rising from 6.5 to 7.2 on average (0-10 scale) from the youngest to the oldest group. We found similarly that average happiness during the task (12 ratings) increased with age ($r = 0.113$, $p < 0.001$), rising from 55 to 61 on average (0-100 scale) from the youngest to the oldest group. Variability in happiness ratings decreased with age ($r = -0.089$, $p < 0.001$), from a standard deviation of 15.4 to 13.6 from the youngest to the oldest group.

Choice consistency in decision tasks can be quantified as the frequency with which participants make the same choice of the safe or risky option when presented with the same options in two trials [S3]. Due to the small number of trials per play, such a metric could not be computed. We instead computed a closely related consistency measure by sorting trials for each trial type according to option value and determining the consistency of choices for adjacent trial pairs in the sorted data.

Computational Modeling

We fitted choice behavior in each individual participant with an approach-avoidance decision model that allowed for value-independent tendencies to choose gambles [S3]. This model accounts for the effects of boosting dopamine with L-DOPA on decision making [S3]. This model also includes the parameters for risk and loss aversion in common parametric decision models [S4-S7] based on prospect theory [S8]. As in those models, subjective values or expected utilities of options were determined using the following equations:

$$U_{gamble} = 0.5\left(V_{gain}\right)^{\alpha_{gain}} - 0.5\lambda(-V_{loss})^{\alpha_{loss}}$$
$$U_{certain} = (V_{certain})^{\alpha_{gain}} \quad if\ V_{certain} \geq 0$$
$$U_{certain} = -\lambda(-V_{certain})^{\alpha_{loss}} \quad if\ V_{certain} < 0$$

where V_{gain} and V_{loss} are the potential gain and loss from a gamble, respectively, and $V_{certain}$ is the certain option value. The degree of curvature in the utility function in the gain domain is determined by α_{gain} and thus the degree of risk aversion for potential reward. An individual that is risk-neutral in gain trials has $\alpha_{gain} = 1$, and would be indifferent between a certain gain and a gain gamble with the same expected value. A risk-seeking individual would have $\alpha_{gain} > 1$ and a risk-averse individual would have $\alpha_{gain} < 1$. Degree of risk aversion for losses is determined by α_{loss}. An individual with $\alpha_{loss} < 1$ would be risk seeking in loss trials and if $\alpha_{loss} > 1$ would be risk averse in loss trials. The parameter λ determines the degree of loss aversion. A gain-loss neutral individual would have $\lambda = 1$ and a loss-averse individual would have $\lambda > 1$.

Choice probabilities in established models [S5, S6] are often determined by the softmax rule:

$$P_{gamble} = \frac{1}{1 + e^{-\mu(U_{gamble} - U_{certain})}}$$

where the inverse temperature parameter μ quantifies the degree of choice stochasticity. The softmax rule maps subjective value differences to probabilities from (0, 1). In our approach-avoidance decision model [S3], we modified the softmax rule to permit choice probabilities that differ from 0 or 1 in the limit. This allows for value-independent effects on choice behavior that might account for Pavlovian influences. Computation of option subjective values was unaffected. We allowed for separate parameters for gain trials and loss trials. For gain trials, the probability of gambling was determined by β_{gain}:

$$P_{gamble} = \frac{(1 - \beta_{gain})}{1 + e^{-\mu(U_{gamble} - U_{certain})}} + \beta_{gain} \quad if\ \beta_{gain} \geq 0$$
$$P_{gamble} = \frac{(1 + \beta_{gain})}{1 + e^{-\mu(U_{gamble} - U_{certain})}} \quad if\ \beta_{gain} < 0$$

For loss trials, the probability of gambling was determined in a similar manner by β_{loss}:

$$P_{gamble} = \frac{(1 - \beta_{loss})}{1 + e^{-\mu(U_{gamble} - U_{certain})}} + \beta_{loss} \quad if\ \beta_{loss} \geq 0$$
$$P_{gamble} = \frac{(1 + \beta_{loss})}{1 + e^{-\mu(U_{gamble} - U_{certain})}} \quad if\ \beta_{loss} < 0$$

If either β parameter is positive, the function maps choice probabilities in that domain from (β, 1). If either β parameter is negative, the function maps choice probabilities in that domain from (0, 1+β). The parameter β acts as a value-independent (but valence-dependent) influence on the probability of gambling.

Model parameters were fit by the method of maximum likelihood for data from individual participants. Economic preference parameters were constrained to the range of values that could be estimated based on the design matrix (λ: 0.5-5, α_{gain}: 0.3-1.3, α_{loss}: 0.3-1.3). We used Bayesian model comparison techniques [S9, S10] to compare fits for the economic decision model and for the approach-avoidance decision model. For each participant, we computed the Bayesian information criterion (BIC), which penalizes for parameter number, and then summed BIC across participants. The model with the lowest BIC is the preferred model.

Supplemental References

S1. Rutledge, R.B., Skandali, N., Dayan, P., and Dolan, R.J. (2014). A computational and neural model of momentary subjective well-being. Proc. Natl. Acad. Sci. USA *111*, 12252-12257.

S2. Mroczek, D.K., and Kolarz, C.M. (1998). The effect of age on positive and negative affect: a developmental perspective on happiness. J. Pers. Soc. Psychol. *75*, 1333-1349.

S3. Rutledge, R.B., Skandali, N., Dayan, P., and Dolan, R.J. (2015). Dopaminergic modulation of decision making and subjective well-being. J. Neurosci. *35*, 9811-9822.

S4. Tversky, A., and Kahneman, D. (1992). Advances in prospect theory – cumulative representations of uncertainty. J. Risk Uncertain. *5*, 297-323.

S5. Sokol-Hessner, P., Hsu, M., Curley, N.G., Delgado, M.R., Camerer, C.F., and Phelps E.A. (2009). Thinking like a trader selectively reduces individuals' loss aversion. Proc. Natl. Acad. Sci. USA *106*, 5035-5040.

S6. Frydman, C., Camerer, C., Bossaerts, P., and Rangel, A. (2011). MAOA-L carriers are better at making optimal financial decisions under risk. Proc. R. Soc. B *278*, 2053-2059.

S7. Sokol-Hessner, P., Camerer, C.F., and Phelps, E.A. (2013). Emotion regulWation reduces loss aversion and decreases amygdala responses to losses. Soc. Cogn. Affect. Neurosci. *8*, 341-350.

S8. Kahneman, D., and Tversky, A. (1979). Prospect theory: an analysis of decision under risk. Econometrica *47*, 263-291.

S9. Schwarz, G. (1978). Estimating the dimension of a model. Ann. Stat. *6*, 461-464.

S10. Burnham, K.P., and Anderson, D.R. (1998). Model Selection and Inference (Springer).
